# Obituary: Hans Bigalke 1946–2024

**DOI:** 10.1007/s00702-025-03075-y

**Published:** 2026-01-22

**Authors:** Dirk Dressler, Jürgen Frevert

**Affiliations:** 1 Movement Disorders Section, Department of Neurology, Hannover Medical School, Carl-Neuberg-Str. 1, 30625 Hannover, Germany; 2 Neurotoxin Research Center, Tongji University School of Medicine, Shanghai, China; 3 Merz Pharmaceuticals GmbH, Frankfurt, Germany

**Figure:**
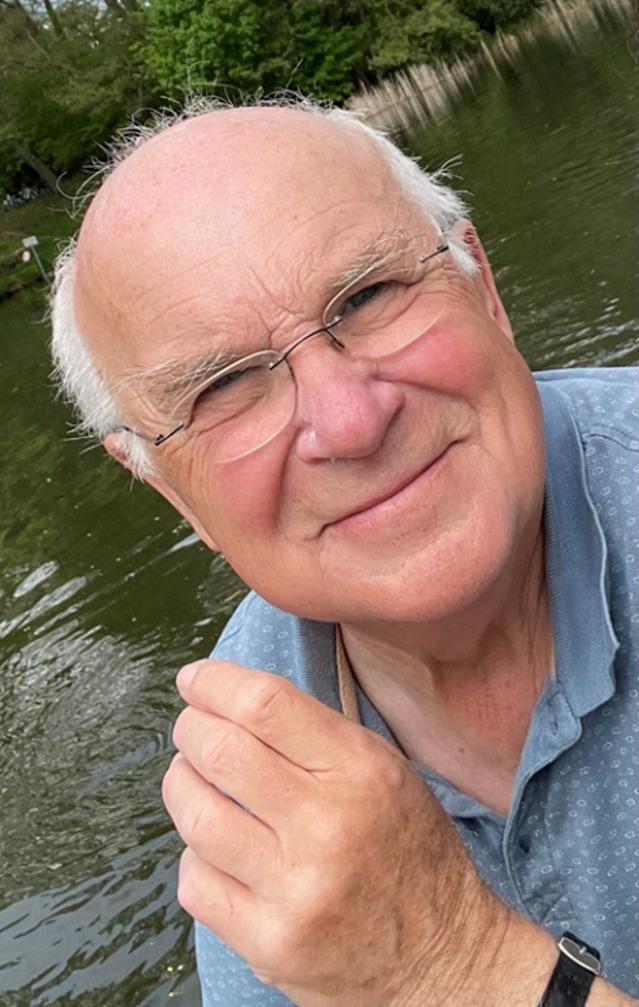
Professor Dr. med. Hans Bigalke 1946–2024.

Professor Dr. med. Hans Bigalke died on October 16th 2024 at the age of 77. He was one of the most important researchers on Clostridium botulinum and botulinum toxins (BT) in Germany and beyond. He combined a brilliant academic career with a keen entrepreneurial spirit.

Hans Bigalke studied medicine at Justus Liebig University in Giessen, Germany. From 1978 to 1980 he completed a postgraduate fellowship with Professor Habermann at the Rudolf Buchheim Institute of Pharmacology and Toxicology in Giessen. Here he started his studies on clostridial toxins by analysing the inhibition of acetylcholine secretion by tetanus and botulinum toxins in primary neuronal cell cultures and isolated brain tissue. In 1981, he joined the laboratory of P. G. Nelson at the National Institutes of Health in Bethesda, MD, USA, to adapt spinal cord cell culturing and electrophysiological techniques to his research on clostridial toxins. Returning to Giessen, he continued his work and developed the mouse hemidiaphragma assay (HDA), an ex vivo assay to replace the mouse lethality assay (LD50 assay) for measuring the potency of BT. In 1986, he habilitated and became Professor of Toxicology and later Vice-Chairman of the Department of Toxicology at Hannover Medical School, Germany.

At that time, BT research was not very popular. Only a few groups in the world were working on food safety and in biodefence on this most poisonous poison. Then, suddenly, the situation changed completely, when Alan B. Scott discovered the BT’s enormous therapeutic potential: first as a powerful tool to treat muscle hyperactivity disorders, including dystonia, spasticity and infantile cerebral palsy, and later to reduce muscular wrinkles. Subsequently, autonomic disorders, including hyperhidrosis and hypersalivation, and even pain disorders such as migraine, became established indications.

In 1997, Hans Bigalke became co-founder and CEO of Toxogen GmbH in Hannover, Germany, where he continued his work on the HDA. He refined the HDA to become a replacement for the LD50 assay for potency testing to release BT drug batches. He also used the HDA to measure accurate titres of neutralising BT antibodies. Eventually, this test was widely used to monitor BT’s therapeutic use, to control clinical trials and to test antigenicity in registration studies of the pharmaceutical industry. In addition, the HDA was a versatile tool for investigating various aspects of BT preparations. In doing so, Hans Bigalke saved the lives and suffering of huge numbers of laboratory mice.

His research also included pivotal work on the role of the complexing proteins naturally associated with botulinum neurotoxin. The demonstration that they are not necessary for BT’s biological and therapeutic effects led directly to the development of the first BT drug without complexing proteins. This novel drug, developed in collaboration with Jürgen Frevert of Merz Pharmaceuticals from Germany, is characterised by particularly low antigenicity and became an internationally successful drug. Research into engineering modifications of BT’s structure led to BT with enhanced receptor binding and to various theoretical models for expanding BT’s future therapeutic use.

Later on, Hans Bigalke became a shareholder and board member of List Biological Laboratories, Campbell, CA, USA, a company also involved in various commercial aspects of bacterial toxins.

Hans Bigalke produced 114 publications (PubMed, 18.02.2025). His main direct collaborators were Andreas Rummel (18 joint publications), Gudrun Ahnert-Hilgert (10 joint publications) and Petra Marxen (7 joint publications). His main external collaborators were the groups of Dirk Dressler at the Department of Neurology at Hannover Medical School (24 joint publications), Thomas Binz at the Institute of Cell Biochemistry at Hannover Medical School (13 joint publications), Reinhard Dengler at the Department of Neurology at Hannover Medical School (8 joint publications) and Harald Hefter at the Department of Neurology at Heinrich Heine University, Düsseldorf (7 joint publications). Further collaborators include Jürgen Frevert (6 joint publications) and Ernst Habermann (6 joint publications). He held several patents on botulinum toxins. In 2023 Hans Bigalke was honoured with the Lifetime Achievement Award of the International Neurotoxin Association.

Hans Bigalke will be remembered by his colleagues, collaborators and students for his kindness and generosity in sharing his vast experience in the field. He is survived by his wife and two daughters.

## Data Availability

No datasets were generated or analysed during the current study.

